# Pharmacokinetic/Pharmacodynamic Modelling of Cefquinome in Lactating Sheep and Lactating Goats After Intravenous, Subcutaneous and Long-Acting Administrations

**DOI:** 10.3390/vetsci13060580

**Published:** 2026-06-13

**Authors:** Carlos Mario Carceles-Rodríguez, Emilio Fernández-Varón, Cristina Bernal Alcaraz, Carlos Cárceles, Rocío Morón-Romero, Xando Díaz-Villamarín, Pilar Muñoz-Rascón, Juan Manuel Serrano-Rodríguez

**Affiliations:** 1 Department of Pharmacology, Faculty of Veterinary Medicine, University of Murcia, 30100 Murcia, Spain; carceles@um.es; 2 Center for Biomedical Research (CIBM), Department of Pharmacology, University of Granada, 18016 Granada, Spain; 3 Emergency and Health Emergencias Service, Servicios Murciano de Salud, 30100 Murcia, Spain; cristina.bernal4@carm.es (C.B.A.); carlos.carceles@carm.es (C.C.); 4 Center for Biomedical Research (CIBM), (Ibs.Granada), Pharmacy Service, San Cecilio Clinical Hospital, 18106 Granada, Spain; rmoronr@gmail.com; 5 Department of Pharmacology and Therapeutics, Faculty of Medicine, Autonomous University of Madrid, 28029 Madrid, Spain; xandodv@gmail.com; 6 Pharmacology Area, Department of Nursing, Pharmacology and Physiotherapy, Faculty of Veterinary Medicine, University of Córdoba, 14014 Córdoba, Spain; v02murap@uco.es (P.M.-R.); jserranor@uco.es (J.M.S.-R.)

**Keywords:** pharmacokinetic/pharmacodynamic, cefquinome, long-acting formulation, lactating sheep, lactating goats

## Abstract

The pharmacokinetics (PK) and pharmacokinetic–pharmacodynamic (PK/PD) relationships of cefquinome in small ruminants remain incompletely characterized, particularly for long-acting (LA) formulations. This study evaluated cefquinome disposition after intravenous (IV), subcutaneous (SC) and LA subcutaneous (SC-LA) administration in lactating sheep and goats using nonlinear mixed-effects models (NLMEs) and Monte Carlo (MC) simulations. Cefquinome exhibited low volumes of distribution (0.21–0.31 L/kg), with goats showing higher clearance and shorter terminal half-lives than sheep. The SC-LA formulation reduced the absorption rate constant and increased both the mean absorption time and terminal half-life by 4–6-fold, resulting in sustained systemic exposure over 48 h. PK/PD analysis showed higher PK/PD cut-off values for the LA formulation, with values of 0.125 μg/mL for the *f*T > MIC index and 0.25 μg/mL for the *f*AUC/MIC index, respectively, whereas IV and SC regimens achieved lower thresholds. MC simulations indicated that only the LA formulation achieved ≥ 90% probability of target attainment (PTA) values at MICs equivalent to tentative epidemiological cut-off values (TECOFF) for respiratory pathogens. Notably, *f*AUC/MIC provided a more informative descriptor of efficacy for the LA formulation. These findings highlight the advantage of LA formulations and demonstrate improved performance compared with conventional dosing regimens in sheep and goats.

## Simple Summary

Cefquinome is a fourth-generation cephalosporin widely used in veterinary medicine, although its pharmacokinetic (PK) properties and optimal dose regimen remain insufficiently characterized in small ruminants. In particular, limited information is available regarding the influence of different formulations on drug disposition and therapeutic effectiveness. This study investigated the PK and the pharmacokinetic–pharmacodynamic (PK/PD) relationships of cefquinome in lactating sheep and goats following intravenous, subcutaneous and long-acting (LA) subcutaneous administration, using nonlinear mixed effect models (NLMEs) and Monte Carlo (MC) simulations as modelling and simulation techniques, respectively. Our findings demonstrate that despite the interspecies differences observed, the LA formulation markedly prolongs drug exposure in both species by slowing absorption and increasing the terminal half-life. This sustained exposure translates into improved probability of target attainment (PTA) against bacterial pathogens. Importantly, the results also suggest differences in the PK/PD index of efficacy: while the percentage of time above the minimum inhibitory concentration (*f*T > MIC) is traditionally used for beta-lactams, the ratio of the area of free concentrations under the curve (*f*AUC/MIC) appears to better describe efficacy for LA formulations. Finally, lower concentrations were observed in milk below the limits of quantification. Overall, these findings highlight that LA formulation may enhance therapeutic outcomes and support more effective and rational antimicrobial use in small ruminants, particularly in the treatment of respiratory infections.

## 1. Introduction

Cefquinome is a broad spectrum fourth-generation cephalosporin developed for veterinary use. The incorporation of a quaternary cation into its beta-lactam structure confers a zwitterionic character, which increases the antimicrobial spectrum, enhances its ability to penetrate the periplasmic space of Gram-negative bacteria, and improves its stability against β-lactamases [1]. This antimicrobial has been approved in the European Union (EU) for the treatment of respiratory tract diseases and septicaemia in pigs, cattle, and horses, as well as for the treatment of mastitis via intramammary administration in cattle. Dose regimen of this drug ranges from 1 to 2 mg/kg each 24 h for 3 to 5 consecutive days, or 2.5 mg/kg every 48 h for a long-acting (LA) subcutaneous (SC) administration in pigs and cattle [2]. However, it is not authorised in sheep and goats, and its administration constitutes extra-label use, which must comply with the regulations established by the European Medicines Agency (EMA) [3]. Consequently, and in accordance with the antibiotic categorisation of the EMA, it belongs to Category B and should only be administered following previous antimicrobial susceptibility testing (AST) [4,5]. Nevertheless, this antibiotic represents an excellent alternative when other treatments fail due to its high efficacy against bacteria such as *Klebsiella pneumoniae* and *Pasteurella multocida* [6,7].

The pharmacokinetics (PK) properties of cefquinome have been described following intravenous (IV), intramuscular (IM), and SC administration of between 2 and 3 mg/kg in non-lactating sheep [7,8]. In goats, PK studies have been conducted with IV and IM administrations in lactating, non-lactating and pregnant animals with similar dose ranges [9,10,11,12,13]. However, SC administration remains uncharacterised in lactating sheep and in lactating and non-lactating goats. In the same way, the PK of LA formulations have not yet been studied in these ruminants, despite the well-recognised advantages that this type of administration offers in veterinary medicine [14,15].

The typical pharmacokinetic/pharmacodynamic (PK/PD) index used for cephalosporins, as time-dependent antibiotics, is the percentage of time that plasma concentration remains above the minimum inhibitory concentration (MIC) during the dosing interval (DI), defined as *f*%T > MIC, with *f* indicating that unbound to plasma protein, or free plasma concentration, should be used to compute this index [16]. To achieve clinical efficacy with beta-lactams, target values of 25–40% and 40–50% of DI for Gram-positive and Gram-negative pathogens are recommended, respectively [17]. However, recent pharmacometrics studies have highlighted that under specific conditions such as prolonged drug exposure, long half-lives or with LA formulations, the different profiles obtained can produce instability and increased variability in the estimation of this index, especially when the Monte Carlo simulation is used. In contrast, the *f*AUC/MIC index, defined as the ratio of the area of free concentration under the curve (AUC) to MIC, typically associated with concentration-dependent antibiotics, integrates drug exposure over time and is primarily driven by clearance and bioavailability, making it less sensitive to the shape of the concentration–time curves [18,19,20]. As a consequence, it may provide complementary information of drug exposure in these scenarios, and recent research recommends its use in combination with the classic *f*%T > MIC [21,22].

For each dose regimen studied, the PK/PD indices must be evaluated against different MIC values to determine the probability of target attainment (% PTA) and their efficacy [17,22]. After this analysis, it is possible to calculate the MIC values at which the desired clinical outcome is achieved, typically defined as the endpoint of PTA at 90%. These MIC values are referred to as PK/PD cut-off values (PK/PD_co_) [23]. Furthermore, the modelling of these data can be performed in a robust and effective way using nonlinear mixed-effects models (NLMEs) [24,25]. This approach has been recommended by the EMA and the European subcommittee for Veterinary Antimicrobial Susceptibility Testing (VETCAST) for conducting Monte Carlo simulations and to optimise dose regimens and to establishment clinical break points in animals (CBPs) [17,26]. Nevertheless, few studies have been conducted on cefquinome using these models [12].

The objectives of this study were to: (1) describe the PK of cefquinome in lactating sheep and lactating goats in plasma and milk following IV, SC and SC-LA administration using a formulation based on Poloxamer P407 (P407) containing 2% of carboxymethylcellulose (P407-CMC); (2) evaluate the absorption and bioavailability of both extravascular formulations in these two ruminant species; (3) determine the efficacy indices and establish the PK/PD_co_ values for different levels of probability of target attainment (PTA); and (4) compare the PK/PD_co_ obtained with the tentative epidemiological cut-off values (TECOFF) established by the VETCAST committee for cefquinome.

## 2. Materials and Methods

### 2.1. Experimental Design, Drug Administration and Sample Collection

The number of animals used in this research was calculated following the indications described for a rich sampling design in PK assays using the Cl published by other studies as the main parameter [7,9,27]. The calculation was developed in the RStudio software (R 4.5.0, R Development Core Team, R Foundation for Statistical Computing, Vienna, Austria).

Six clinically healthy Lacaune lactating sheep (83.5 ± 7.9 kg), aged from 2.5 to 3.5 years, from a private sheep farm in Albacete, Spain, were used. Six clinically Murciano-Granadina lactating goats (48.5 ± 10.9 kg), aged from 2.5 to 3.5 years, from the Caprine Farm of the University of Murcia were used. The animals were housed and fed an antibiotic-free diet for at least 30 days prior to the study. Alfalfa hay and water was provided ad libitum together with a drug-free concentrate.

A cross-over design (Latin square 3 × 3) was used in three phases for both ruminant species. Each animal received either a single IV or SC injection of cefquinome sodium (Vetranal^®^, batch: 9147403, Sigma-Aldrich, Madrid, Spain) at a 2 mg/kg dose or an SC-LA administration of 6 mg/kg with at least a 15-day washout period.

For the IV administration, the solution was injected into the left jugular vein, and blood samples (4 mL) were collected from the contralateral jugular vein. SC and SC-LA injections were administered under the skin of the back at a single location in the thoraco-lumbar region lateral of the mid-line. The dose was selected for LA formulation.

Blood samples were collected at 0 (pre-treatment), 0.083, 0.167, 0.25, 0.5, 0.75, 1, 1.5, 2, 4, 6, 8, 10, 12, 24, 32, 48, and 72 h post-dosing. Samples were centrifuged at 1500 *g* for 15 min, and the plasma was taken and stored at −80 °C until assayed. Milk samples for analysis were collected from each animal after complete evacuation of the udder by manual stripping of each gland immediately before dosing on the day of treatment administration (time 0) and at 1, 2, 4, 6, 8, 10, 12, 24, 32, 48, and 72 h after administration. After shaking the milk to homogenize, a 4–5 mL sample was collected and stored at −80 °C until assayed.

### 2.2. Gel Preparation for LA Formulation

In the present study, an aqueous polymeric sustained release formulation of P407 with 2% of CMC was prepared on a weight basis using the cold method [14,28,29]. Concentrations of P407 (BASF, Barcelona, Spain), CMC sodium (Sigma-Aldrich, Madrid, Spain) and cefquinome sulphate (Vetranal, Fluka analytical, Sigma-Aldrich, Madrid, Spain) reported in this assay are expressed as weight/volume percentage (% wt/v). An amount of P407 that was sufficient to yield 25% and CMC to yield 2% gel was slowly added at 4 °C and of cefquinome sulphate that was sufficient to yield a 20% concentration was dissolved in the cold solution. The dispersion was refrigerated until a clear solution was formed (6–12 h).

The dose selected for the SC-LA formulation (6 mg/kg) was based on previous studies using poloxamer P407-based delivery systems [19,28]. These formulations are characterised by slow and sustained drug release from the polymeric matrix, and higher doses are commonly required to maintain plasma concentrations within the therapeutic range over extended dosing intervals [14,19,29].

### 2.3. Analytical Method

Plasma and milk concentrations were measured using high-performance liquid chromatography (HPLC) methods previously described with ultraviolet detection and with cefotaxime as an internal standard [7,30]. A volume of a sample of 200 µL of plasma or milk was mixed with 100 µL of methanol and 100 µL of a trifluoroacetic acid/methanol solution (1:2). They were then subjected to shaking and sonication for 5 min followed by centrifugation for 10 min at 10,000 rpm (14,000 in the case of milk). From the supernatant, 300 µL were extracted and transferred to the HPLC autoinjector vials, and 100 µL were injected. The separation was performed using an ULTRABASE 100 Å 5 μm, 250 mm × 4.6 mm reversed-phase column (Altmann Analytik GmbH & Co. KG, Múnich, Germany)and ODS (C18) TECNOKROMA 10 × 3.2 mm^2^ guard column (Altmann Analytik GmbH & Co. KG, Múnich, Germany). The samples inside the autoinjector (Peltier system) and the column were thermostatted at 25 °C. The mobile phase consisted of acetonitrile (16%) and trifluoroacetic acid in a 0.1% solution (86%). The flow rate was 1 mL/min. Detection was carried out at a wavelength of 268 nm.

Quality controls were prepared from a pool of blank goat or sheep plasma or milk spiked with seven concentrations of cefquinome of between 0.1 and 10 μg/mL. Aliquots were extracted, and 100 μL were injected into the chromatographic system. Standard curves were obtained by unweighted linear regression of cefquinome and cefotaxime peak areas versus known concentrations. Each point was established from an average of five determinations. Correlation coefficients (r) were >0.99 for calibration curves. The percentage recovery was determined by comparing the peak areas of plasma and milk blank samples spiked with different amounts of the drug (0.25, 1.0 and 5.0 µg/mL) and treated as any samples, with the peak areas of the same standards prepared in a phosphate buffer. Each point was established from an average of five determinations. In sheep, the mean percentage recoveries of cefquinome from plasma and milk were 100.83 and 100.23%, respectively, and 98.25 and 97.14%, respectively, in goats. The assay precision (R.S.D.) was assessed by expressing the standard deviation of repeated measurements as a percentage of the mean value. Intraday precision was estimated from 6 replicates of 3 standard samples used for calibration curves. Intraday precision was R.S.D. < 10% (plasma) and R.S.D. < 14% (milk). Interday precision was estimated from the analysis of standard samples (plasma or milk) on 3 separate days. Plasma interday obtained an R.S.D < 12%. Milk interday assay obtained an R.S.RD < 14%. The limit of quantification (LOQ) and the limit of detection (LOD) was 0.05 μg/mL for plasma and milk.

### 2.4. Pharmacokinetic Modelling and Statistical Analysis

Plasma concentrations of cefquinome, determined by the HPLC method, were corrected according to the free unbound fraction previously published, with values of 0.8435 for sheep and 0.8560 for goats, respectively [10]. Only free concentrations were used for the PK analysis, and all estimated parameters and PK/PD ratios calculated in this study incorporate this correction [31]. Moreover, milk concentrations were not included because they were found to be below the LOQ in goats, and in the case of sheep, only low concentrations were detected (up to 3.0 µg/mL) in a few samples and animals between 2 and 12 h post-administration. Therefore, these data were insufficient for inclusion in the modelling.

Free plasma concentration profiles in sheep were modelled independently from those generated in goats. Moreover, from each ruminant group, data from IV, SC and SC-LA formulations were simultaneously analysed by NLME modelling with the MonolixSuite 2024R1^®^ software (Simulations Plus/Lixoft, Ltd., Lancaster, CA, USA). In the first step, several models were explored with different compartments and absorption profiles. In the second step, the final model was selected based on a reduction in variability and the evaluation of multiple statistical criteria, including the likelihood ratio test (−2·log-likelihood, −2LL), Akaike Information Criterion (AIC), and Bayesian Information Criterion (BIC) [32]. Each model studied was also assessed through graphical diagnostics, including goodness-of-fit plots (observed versus population/individual predictions), weighted residual plots (PWRES and IWRES versus time or predictions), and visual predictive checks plots (VPCs) [33].

**Figure 1:**
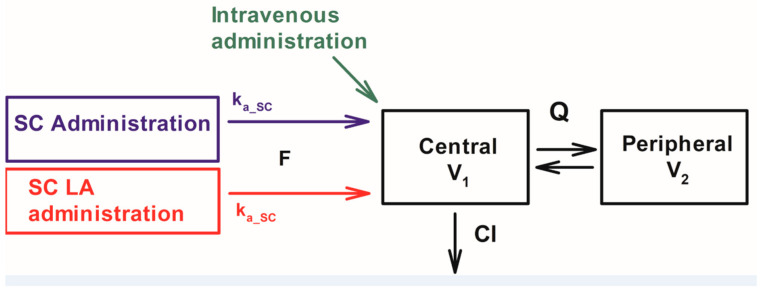
Diagram of the model selected to describe the simultaneous disposition of cefquinome in lactating sheep and lactating goats after intravenous and subcutaneous administration using two extravascular formulations.

The disposition of the drug was described with a model with two compartments, central and peripheral, with linear elimination from the central compartment. The absorption process was time dependent and described by a Weibull function [34]. This model was applied for both the sheep and goat datasets and was written as an ordinary differential equation system and is shown in Equations (1)–(4):

(1) $$\frac{dA_{1}}{dt}\;=\;F\cdot WF\cdot A_{3}-\;\frac{Cl}{V_{1}}\;\cdot A_{1}-\;\frac{Q}{V_{1}}\cdot A_{1}+\;\frac{Q}{V_{2}}\cdot A_{2}$$

(2) $$\frac{dA_{2}}{dt}=\frac{Q}{V_{1}}\cdot A_{1}-\frac{Q}{V_{2}}\cdot A_{2}$$

(3) $$\frac{dA_{3}}{dt}=-F\cdot WF\cdot A_{3}$$

(4) $$WF=\left(k_{a}\cdot \gamma \right)\cdot {\left(k_{a}\cdot t\right)}^{\gamma -1}$$

where A_1_, A_2_ and A_3_ are the amounts of cefquinome in central, peripheral and depot compartments, respectively. Plasma concentrations were calculated as C_1_ = A_1_/V_1_. F is the SC bioavailability, expressed as a fraction between 0 and 1; WF is the Weibull function, which describes the absorption processes that do not follow first-order kinetics and is parameterized by k_a_ (the absorption rate constant) and gamma (the shape parameter that influences the slope of the absorption phase); V_1_ is the volume of distribution at the central compartment; Cl is the clearance; Q is the intercompartmental clearance; and V_2_ is the volume of distribution at the peripheral compartment. Figure 1 shows a schematic representation of the PK model developed.

In sheep, the residual unexplained variability (RUV) from the predicted concentrations was defined using a combined error model described as C_obs_ = C_pred_ + (a + b∙C_pred_)∙ε, whereas in goats, a proportional error model was selected and described as C_obs_ = C_pred_ + b∙C_pred_∙*ε*, where C_obs_ is the observed plasma concentration, C_pred_ is the predicted concentration, and a and b are components for the residual error [33]. However, it is important to note that the RUV error in NLME modelling not only reflects analytical variability but also intra-individual variability and potential model misspecification [32]. This selection was based on residual diagnostics and goodness-of-fit evaluations, with no evidence of systematic bias observed in the final models [24].

The variability for each parameter of the model was assumed to be log-normally distributed across the data, whereas that for SC bioavailability assumed a logit-normal distribution to bound predictions between 0 and 1 as follows (Equation (5)):

(5) $$\theta_{i}=\theta_{\mathrm{TV}}\;\cdot e^{\eta_{IIV}}\cdot e^{\eta_{IOV}}$$

where θ_i_ is the parameter estimate for each ith subject from the dataset, θ_TV_ is the typical value estimated by the model, η_IIV_ is the inter-individual variability (IIV), and η_IOV_ is the inter-occasion variability (IOV) associated with the difference between occasions [32,33].

The influence of two covariates was studied in the disposition of cefquinome: the weight of animals as a continuous covariate and the type of formulation as a categorical covariate (conventional SC versus SC-LA formulation). Both were tested with the method of conditional sampling for a stepwise approach based on correlation tests, the Pearson correlation test, the Wald test and the analysis of variance (ANOVA) [35]. Furthermore, it was also studied whether these covariates reduced the values of IIV, IOV, −2xLL, AIC, and BIC [32,33]. After this analysis, only the effect of the formulation was included as a categorical covariate, and Equation (5) was modified to Equation (6), with Cov_θi_ as the exponent for the covariate effect (Equation (6)):

(6) $$\theta_{i}=\theta_{\mathrm{TV}}\cdot e^{\eta_{IIV}}\cdot e^{\eta_{IOV}}\cdot e^{{Cov}_{\theta i}}$$

The robustness of the model was verified using a convergence assessment in Monolix (*n* = 500). The eta shrinkage (η) values for each typical estimated parameter were computed. Non-parametric bootstrap analysis was developed at the 95% confidence interval of parameter estimates (200 replicates) [36]. The code of the final models is included in the Supplementary Materials.

The secondary parameters obtained were the following: AUC_0_^∞^, the area under the concentration–time curve from zero to infinity, calculated directly by integration of the model; C_max_, the maximum plasma concentration following extravascular administration; T_max_, the time to reach C_max_; V_ss_, the volume of distribution at steady state; t_1/2_, the half-life associated with the elimination phase; MRT, the mean residence time; and MAT, the mean absorption time after extravascular administration.

Descriptive statistics and distribution tests were performed for all PK parameters obtained. The distribution of the data was normal, and parametric tests were used. The ANOVA test was used to compare differences between parameters. When significant differences were found, a *t*-test was used as a second post hoc test (pairwise or un-pairwise comparison). A Bonferroni correction was applied for this *t*-test. The significance level was set at *p* < 0.05. This analysis was performed in Rstudio with R version 4.5.0 (R Development Core Team, R Foundation for Statistical Computing, Vienna, Austria).

### 2.5. Simulation and Monte Carlo Analysis

The concentrations and parameters estimated by the model were used to perform two different Monte Carlo simulations in Simulx^®^ (Simulations Plus/Lixoft, Ltd., Lancaster, CA, USA). First, simulated dose regimens in sheep and goats were built in two scenarios (*n* = 10,000 per group); IV and SC routes were done at 2 mg/kg every 24 h until 3 days of treatment (72 h), whereas for LA formulations a single 6 mg/kg dose for 3 days was produced at 72 h. Second, these simulated dose regimens were used to calculate the PK/PD indices. While *f*%T > MIC represents the classical index for β-lactam antimicrobials, *f*AUC/MIC was also considered in the present simulation due to its reported robustness in scenarios involving prolonged drug exposure, such as LA formulations or absorption-limited kinetics [17,22]. The use of both indices allowed for a comprehensive evaluation of antimicrobial exposure and a comparison of their influence on the simulations [20].

For the *f*%T > MIC index, a DI of 40% was selected for each dose regimen and formulation (9.6 h for IV and SC and 29 h for SC-LA administration). For the *f*AUC/MIC index, simulated AUC_24_ and AUC_72_ values were used to calculate the *f*AUC_24_/MIC ratios for IV and SC formulations and *f*AUC_72_/MIC ratios for LA formulations, respectively [17]. Finally, each PK/PD index obtained was plotted against an MIC range from 0.008 μg/mL to 8.0 μg/mL, and the PTA was obtained. The highest probability selected was 90% [37].

After these simulations, different PK/PD_co_ values were obtained for sheep and goats for each dose regimen, formulation and PK/PD index. These values were compared with the published TECOFF of cefquinome against pathogens such as *Actinobacillus pleuropneumoniae*, *K. pneumoniae*, *Mannheimia haemolytica* and *P. multocida* from the database of the European Committee on Antimicrobial Susceptibility Testing (EUCAST) [38,39].

## 3. Results

Clinical examination of all ruminants after each phase of the trial did not reveal any abnormalities. No local or systemic adverse reactions were observed after IV, SC, and SC-LA administration.

### Pharmacokinetic and Statistical Analysis

**Figure 2:**
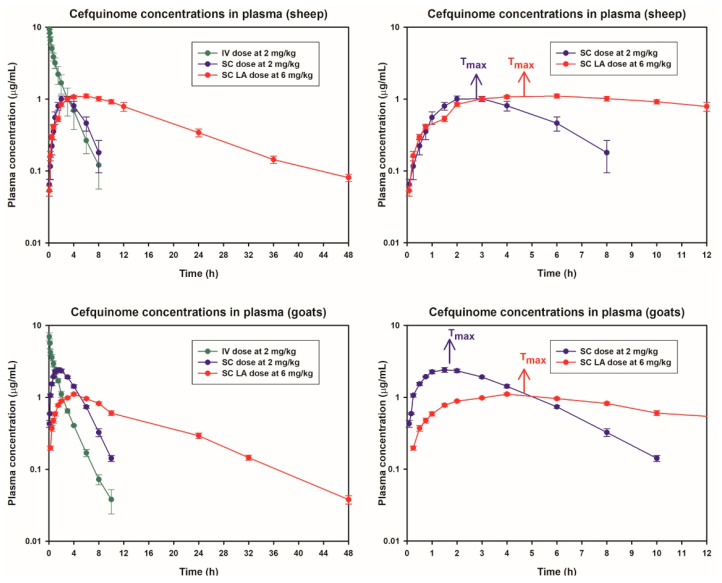
Semilogarithmic plot of plasma concentrations (mean ± SD) of cefquinome. (**Top left**): IV administration at 2 mg/kg (green), SC administration at 2 mg/kg (blue), and SC-LA administration at 6 mg/kg (red) in lactating sheep. (**Top right**): Plasma concentrations in the first 12 h after SC administration at 2 mg/kg (blue) and SC-LA administration at 6 mg/kg (red) in lactating sheep. (**Bottom left**): IV administration at 2 mg/kg (green), SC administration at 2 mg/kg (blue), and SC-LA administration at 6 mg/kg (red) in lactating goats. (**Bottom right**): Plasma concentrations in the first 12 h after SC administration at 2 mg/kg (blue) and SC-LA administration at 6 mg/kg (red) in lactating goats.

The mean and standard deviation (SD) of cefquinome concentrations is shown in the upper left and right panels of Figure 2 for sheep and goats, respectively. IV and SC administration at 2.0 mg/kg are displayed in green and blue lines, respectively. Concentrations after SC-LA administration at 6 mg/kg are plotted in red. The lower left and right panels describe the SC and SC-LA absorption profiles during the first 12 h, including the C_max_ and T_max_ values to facilitate comparative analysis between absorption curves.

**Figure 3:**
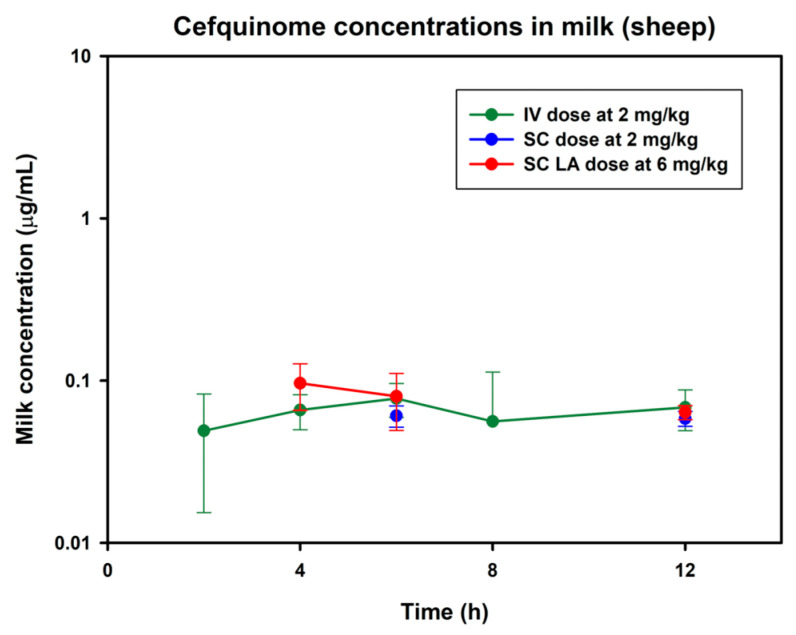
Milk concentrations of cefquinome in sheep after intravenous (red lines), subcutaneous (blue lines) and long-acting subcutaneous administration (red lines).

Table 1 summarises the parameters obtained from the modelling of the data for sheep and goats. Figure 3 shows the milk concentrations of cefquinome obtained after intravenous, subcutaneous and LA administration. As can be observed, the concentrations obtained were very low and insufficient to be included in the PK modelling step. Similarly, the concentrations in goats were below the LOQ and were therefore not included in the analysis. These findings are consistent with previous studies reporting that cefquinome excretion into milk following systemic administration is very low [10,12].

Although the same analytical methodology was used for both ruminant species, the residual unexplained variability observed in the models does not exclusively represent an assay-related error. Instead, it reflects a combination of analytical variability, intra-individual variability, and potential structural model limitations [24]. Therefore, the use of different residual error structures between species likely reflects differences in data dispersion and variability patterns rather than purely analytical noise [32].

**Figure 4:**
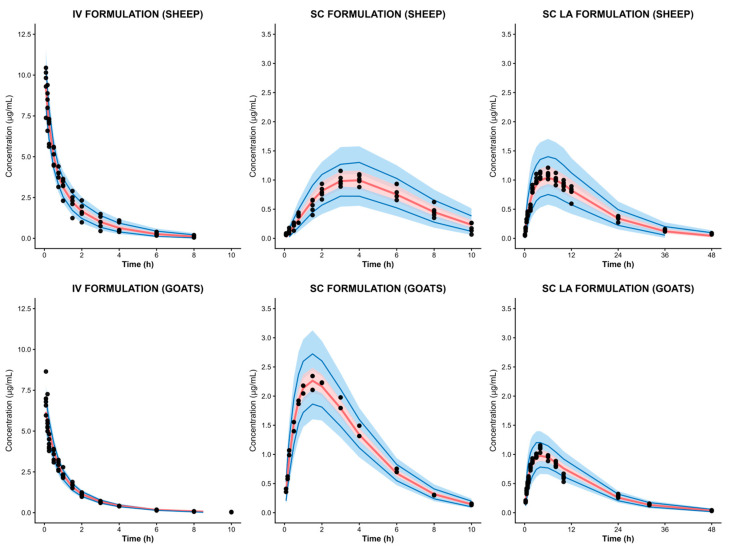
Visual predictive check of plasma concentrations of cefquinome in lactating sheep and lactating goats stratified by route and formulation. Observed concentrations are shown in black. Plots are presented as prediction intervals, with the 10th and 90th percentiles in blue and the 50th percentiles in pink (median). The empirical percentiles of the observed data are displayed as blue lines and median as pink lines.

The VPC plots for each route and formulation in both ruminants are displayed in Figure 4, and it can be observed that most observed data (black points) fell within the prediction intervals (blue and pink areas) and were centred around the median (50%). The precision of the parameters was good (CV ≤ 25% in most estimates), with shrinkage values from −7.28% to 39.2%, indicating adequate distribution of individual parameters throughout the population [40]. The plots of observations versus predictions, residuals, and bootstrap analysis suggest a good description of the observed data (Tables S1 and S2 of the Supplementary Materials). Secondary parameters are shown in Table 2, and statistical comparisons between parameters are described in Table S3 of the Supplementary Materials. In this way, diagnostic and covariate plots support these observations (Figures S1–S6, Supplementary Materials).

The distribution of the drug was low, with volume of distributions that did not exceed unity (0.21–0.31 L/kg, Table 1 and Table 2) and with higher values in sheep (Table S3). These findings suggest that cefquinome, similar to other cephalosporins, presents a distribution process limited principally to the extracellular fluid, as has been previously reported [7,9,30]. Plasma clearance was low, with an overall extraction ratio of 0.04 and 0.02 in sheep and goats, respectively. Lower values were observed in goats, indicating that this antimicrobial can be classified as a drug with low clearance in small ruminants, based on previously established veterinary break points [41].

Terminal half-lives for IV and SC were close to 1.4 to 1.8 h in sheep and goats (Table 2). However, the highest values were observed with the LA formulation and were close to 10 h for sheep and 7.6 h for goats and were significantly different from a statistical point of view between extravascular routes and species, with longer values in sheep (Table S3). The same differences were found in MRT, T_max_ and MAT (Table S3).

The SC bioavailability of cefquinome was moderate, with values of 64% in sheep and 46% in goats and with higher values in sheep but with no differences between formulations in each specie (Table 1 and Table S3). However, the absorption process was influenced by the formulation, as reflected by the k_a_ values in sheep and goats and by the gamma parameter in sheep (Table 1 and Table 1 and Table S3). These results indicate a slower absorption for the LA formulation compared with the conventional SC formulation, as evidenced by their higher T_max_ and MAT values observed (Figure 2, Table 1 and Table 1 and Table S3). The C_max_ values were not different between sheep but were higher in goats after SC administration (Table S3).

## 4. PK/PD Indices and Monte Carlo Simulations

**Figure 5:**
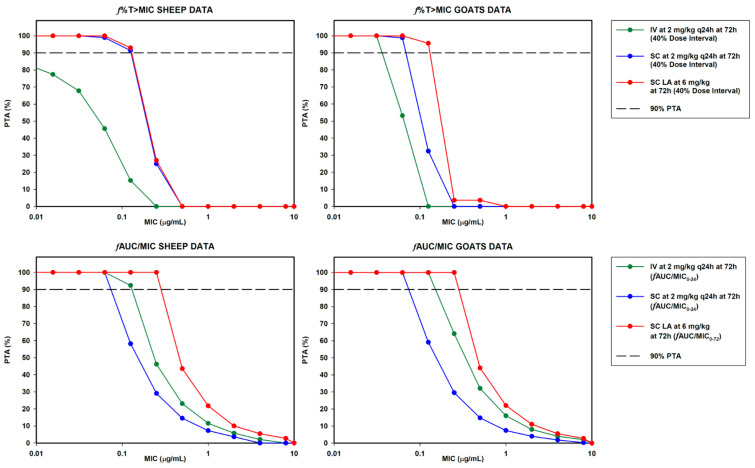
Probability of target attainment values (PTA, %) of cefquinome in plasma of lactating sheep and lactating goats versus MIC. Values obtained for IV, SC and SC-LA administrations after Monte Carlo simulations with different PK/PD indices; *f*%T > MIC and *f*AUC/MIC. Each index is shown as the mean and displayed in green, blue and red lines for IV, SC and SC-LA administrations, respectively. The PTA value of 90% is plotted as the black dashed line.

For each dose regimen simulated, the PK/PD indices provided consistent PK/PD_co_ values across formulations and species, although differences in variability and sensitivity to the plasma profile were observed. These results are described in Table 3, and the PTA values over the MIC are plotted in Figure 5.

For the time-dependent index *f*%T > MIC in lactating sheep and for the IV administration, the values were below the cut-off corresponding to a 90% PTA and could not be determined (Figure 5), whereas for the two extravascular routes, both were 0.125 μg/mL. In lactating goats, these values were 0.03, 0.06, and 0.125 μg/mL for the IV, SC, and SC-LA formulations, respectively. The PTA curves show different profiles for IV and SC routes between sheep and goats (Figure 5).

For the concentration-dependent index *f*AUC/MIC, the same PK/PD_co_ values were observed between lactating sheep and lactating goats (Table 3), with concentrations of 0.125, 0.0625 and 0.25 μg/mL for IV, SC and SC-LA administrations, respectively. This shows close similar PTA curves for each dose, route and formulation (Figure 5).

The TECOFF values for cefquinome against relevant respiratory pathogens were obtained from the EUCAST MIC distribution database (https://www.eucast.org, accessed on 10 December 2025). Reported TECOFF values ranged from 0.06 to 0.125 µg/mL for key pathogens, including *A. pleuropneumoniae*, *M. haemolytica*, *P. multocida*, and *K. pneumoniae*, providing a reference framework for the interpretation of the PTA in the present study. These values were used to contextualise the PK/PD_co_ values derived from Monte Carlo simulations.

## 5. Discussion

In this study, the PK of cefquinome was evaluated in lactating sheep and lactating goats following IV, SC, and SC-LA administrations using NLME models. The results obtained were used to calculate the PK/PD indices %*f*T > MIC and *f*AUC/MIC and to perform Monte Carlo simulations in order to determine the %PTA at different dose regimens, thereby assessing the PK/PD cut-off values and comparing them with the published TECOFF values for cefquinome. In main lines, the differences observed in the results can be attributed to the PK properties of the formulations used for each animal species, whereas the PK/PD_co_ obtained after simulations influenced both the PK properties and the PK/PD index selected.

After IV administration, the volume of distribution showed species-related differences (Table S3). In sheep, the V_ss_ obtained (0.31 L/kg) fell in the previously reported values of 0.21 to 0.36 L/kg in non-lactating sheep [7,8,42]. In goats, however, the computed V_ss_ of 0.21 L/kg was lower than the results reported by other groups of 0.26–0.44 L/kg in lactating and non-lactating sheep [9,12,30,43]. Differences between breeds, HPLC assays or PK modelling of the data may account for these discrepancies [44,45]. Overall, our findings are consistent with the moderate distribution expected for a hydrophilic β-lactam antibiotic with low plasma protein binding [12,30].

In sheep, the clearance estimated (0.18 L/h/kg) was similar to the reported data from other studies with this specie [8,42] and was higher than the value observed in goats of 0.12 L/h/kg (Table S3). However, our clearance in goats was higher than the values described by other authors of 0.04 to 0.068 L/h/kg in lactating and non-lactating goats [9,30,43]. Moreover, these data were lower than the value of 0.294 L/h/kg reported in non-pregnant, pregnant, and lactating goats [12]. Similar to the volume of distribution, these differences may be attributable both to methodological variation across studies and to the breed used, as our research is the only one conducted in Murciano-Granadina goats. Whether these differences could be a key factor in goats remains unclear. However, it has been reported that the status of the animals can influence the disposition of cefquinome in goats [12]. Consequently, further studies could be necessary to evaluate this hypothesis.

The terminal half-life of cefquinome following IV dosing in sheep of 1.66 h was closely related with previous values reported [7,8,42]. In goats, however, the value estimated in our study of 1.44 h was shorter than those reported in several investigations, with values between 4.5 and 6 h in healthy animals [9,30,43]. Finally, after comparisons in our research, the terminal half-life was similar between sheep and goats from a statistical point of view (Table S3), possibly because the differences in V_ss_ and Cl offset one another [44]. At this point, is necessary to take into account that the half-life is a secondary parameter highly sensitive to analytical methodology, PK approaches, and the breed or species used in each study. Consequently, a comparison of this parameter must account for these sources of variability [44].

The absorption of cefquinome after extravascular administration was described using a Weibull function, a useful approach for characterising nonlinear absorption processes and formulation modulated release profiles [34,46]. The covariate analysis accurately differentiated the influence of the formulation in this function, which reduced k_a_ values in both species for SC-LA administration in comparison to conventional SC administration: from 0.25 to 0.072 1/h in sheep and from 0.53 to 0.077 1/h in goats (Table 1). Both reductions were close to 70–85%, suggesting a slow release in which absorption becomes the rate-limiting process [47]. In this context, the secondary parameters supported these observations (Table 2). In fact, the MAT and t_1/2_ values were increased by 4.5–6- and 5–6-fold in comparison to conventional SC administration, suggesting a prolonged exposure [19]. T_max_ values from SC-LA formulations were 1.4- and 2.3-times higher than values obtained after SC administration (Table 3), but the C_max_ values were not different in sheep despite the use of a higher dose by threefold (Figure 2, Table 2 and Table S3).

The evaluation of cefquinome absorption reflects that this antibiotic was probably eliminated from plasma after SC-LA administration at a slower rate than after IV or conventional SC administration, which could be indicating the presence of a ‘flip-flop’ effect characterized by differences in MAT and MRT, as well as the reduced k_a_ values estimated [44,47]. In main lines, these observations indicate that this formulation produced sustained absorption and a marked prolongation of exposure in both species, consistent with previous studies describing similar effects in rabbits and goats with P407-based formulations [19,28,48,49]. Moreover, the higher dose administered in the SC-LA group (6 mg/kg) is aligned with previous studies employing poloxamer P407-based systems [19,29]. These in situ-forming depot formulations often require increased nominal doses to compensate for delayed absorption and to ensure that therapeutic concentrations are maintained throughout the intended dosing interval [14,15,29]. Nevertheless, the absence of a dose-matched control group could represent a limitation when attempting to fully separate formulation-related effects from potential dose-dependent influences. Although cefquinome is generally characterised by linear pharmacokinetics within the therapeutic range, and to our knowledge, there is no evidence suggesting saturable elimination in ruminants, a minor contribution of dose-related effects cannot be completely excluded.

A marked difference in C_max_ between species was observed following SC administration, with goats exhibiting significantly higher peak concentrations than sheep (Table 2 and Table S3). Under conventional conditions, drug absorption is primarily governed by physiological factors at the injection site, including local blood flow, tissue perfusion, and subcutaneous composition, all of which may differ between species and influence the rate and extent of drug uptake [47]. In contrast, this difference disappeared when the SC-LA formulation was administered with comparable C_max_ values between species. This behaviour can be explained by the nature of the poloxamer P407-based delivery drug system [15,29]. As a result, peak concentrations tend to be attenuated and less sensitive to interspecies variability, leading to the convergence of C_max_ values observed in the present study. Similar behaviour has been described for other LA formulations, where controlled release mechanisms override physiological variability at the absorption site and promote more uniform systemic exposure profiles across different populations [50].

In the present study, cefquinome concentrations in milk were below the LOQ in goats, and only residual values up to 3 µg/mL were detected at some data points between 2 and 12 h in sheep (Figure 3). To our knowledge, this is the first study to characterise the disposition of cefquinome in lactating sheep, and the low milk concentrations detected are consistent with previous findings in lactating goats reporting minimal milk excretion and support the observation that transfer into the mammary gland of this drug is extremely limited due to the hydrophilic and zwitterionic properties, which restrict its penetration into mammary tissue [12,30]. These results are consistent with those reported by Shpigel et al., 2006 [51], where intramuscular administration of cefquinome in cows with subclinical *Staphylococcus aureus* mastitis did not improve therapeutic outcomes compared to the spontaneous cure rate observed in untreated controls.

The PK/PD analysis, based on PK/PD_co_ values obtained from the MC simulations, revealed differences between species, PK/PD indices and formulations. In both ruminants, the IV and conventional SC simulated dose regimens showed limited efficacy to reach the target at 90% of PTA, achieving low PK/PD_co_ values, with the exception of the SC regimen in goats with the *f*%T > the MIC index and IV regimen with *f*AUC/MIC in sheep (Tables S4 and S5 of the Supplementary Materials). As a result, these formulations only maintained a PTA ≥ 90% at low MICs, whereas that of the LA formulation displayed a different profile in both species (Figure 5). The extended absorption phase increased the terminal half-life (Table 2) and improved the attainment of PK/PD thresholds (Figure 5). As a consequence, the PK/PD_co_ values were higher for the LA formulation, achieving higher MICs over the same 48 h period until 0.125 µg/mL for *f*%T > MIC and 0.25 µg/mL for *f*AUC/MIC (Table 3). This difference regarding the PK/PD index applied is important. In fact, in both sheep and goats, the PK/PD_co_ based on *f*AUC/MIC was twice that derived from *f*%T > MIC, indicating greater robustness of the LA formulation against less susceptible pathogens. This outcome is consistent with the prolonged systemic exposure generated by this formulation. Nevertheless, it should be emphasized that both indices led to consistent conclusions regarding the PTA achievement, supporting the robustness of the results. Importantly, the use of *f*AUC/MIC should not be interpreted as a replacement of the classical *f*%T > MIC index but rather as a complementary and, in certain contexts, more stable descriptor of antimicrobial exposure under specific conditions, such us under LA formulations [28,52,53].

The comparison of PK/PD_co_ values derived from Monte Carlo simulations with the TECOFF values allows for a clinical assessment of the different formulations in small ruminants (Table 3). The dose regimen computed with the IV and conventional SC formulation achieved limited PK/PD_co_ values. Consequently, it could be suitable only for pathogens with low MIC distributions, such as *A. pleuropneumoniae* and *M. haemolytica*. On the other hand, the higher PK/PD_co_ values obtained with the LA formulation, and especially using the *f*AUC/MIC ratio, indicate that it could achieve pathogens such as *P. multocida* or *K pneumoniae* with higher TECOFF values (Table 3). The rightward shift in the PTA curves (Figure 5) illustrates this enhanced PTA for the LA formulations under use of this index as a tentative predictor of efficacy. Similar findings have been found with other drugs, such as tulathromycin, ceftiofur and florfenicol, suggesting the relevance of *f*AUC/MIC as a pharmacodynamic index of efficacy when LA formulations are used [19,20,21,22,54]

Species differences also contributed to the achieved outcomes. The sheep values were slightly higher than goats for IV and SC regimens, consistent with their lower clearance (Table 1). Nevertheless, the long-acting formulation achieved the same PK/PD values for each PK/PD index in both species (Table 3). Overall, these findings indicate that this type of formulation based on the P407 gel substantially could expand the therapeutic potential of cefquinome against different respiratory pathogens, as has been described with another related drug, ceftiofur, in lactating goats [19].

The findings described in this study are based on healthy lactating ruminants, but the effects on the disposition of a drug in unhealthy animals can be influenced by the disease status because there is evidence that inflammation and infection can modify PK properties [12]. In fact, a decrease in protein binding, downregulations of CYP-450 enzymes or overexpression of transporters can alter the distribution and elimination processes [54]. In this context, it has been demonstrated that induced endotoxemia in sheep significantly decreased the clearance and increased the half-life through changes in perfusion and systemic inflammation in non-lactating sheep and non-lactating goats [42,43]. However, for lactating goats with induced *S. aureus* mastitis the opposite was found, with an increase in clearance in unhealthy animals [30]. These observations indicate that infection-induced physiological changes can influence PK/PD target attainment. Consequently, cefquinome dosing may need adjustments in diseased animals, particularly when the infection stage or systemic inflammation increases the PD requirements. This point was demonstrated using a mouse pneumonia model, where the efficacy of cefquinome against *P. multocida* required higher PK/PD targets at advanced infection stages [55,56]. In this way, the prolonged exposure achieved with LA formulations used in our research may therefore provide a therapeutic advantage in field respiratory infections, typically characterised by high bacterial inocula [20,21].

The analysis performed in this research with lactating animals was based on systemic exposure, and the derived PK/PD indices and PK/PD_co_ values were linked to plasma concentrations and MIC values of respiratory pathogens. However, it is acknowledged that lactation may influence drug disposition, but the magnitude of this effect can vary across studies and species [12,18,19,30]. In a recent study, Litterio et al., 2020 [13] demonstrated that PK/PD evaluations based on plasma concentrations can be reliably applied in lactating and non-lactating animals when targeting systemic infections, such as respiratory diseases. Similarly, El Badawy et al., 2015 [30] reported limited systemic exposure after intramammary administration with low drug concentrations in plasma, reinforcing the concept that the lactation status has limited relevance for systemic effects of cefquinome. Nevertheless, the findings obtained in the present study should be extrapolated to non-lactating populations with caution, and further studies are warranted to determine whether lactation may have influenced the PK/PD results generated by our model.

Finally, it is necessary to indicate that there are several limitations in this study. First, this research was developed with healthy lactating animals, but as previously indicated, the effect of diseases on drug disposition in sheep and goats should be considered in further studies as well as in non-lactating animals [55]. Second, only six animals were used in a crossover design, which is statistically appropriate for a PK preclinical study with intensive sampling [27], but higher animal populations should be studied in a clinical context. Third, the PK/PD_co_ values obtained were influenced for each PK/PD index selected, and, due to the fact that there were no established typical targets for the *f*AUC/MIC index with beta-lactam antibiotics, unlike the well-defined threshold for *f*%T > MIC, it is difficult to determine which PD target could be better. However, our results suggest that *f*AUC/MIC may provide a more appropriate descriptor of efficacy, particularly when LA formulations were used [53,57]. Nevertheless, the results derived from this study offer important insights into the prudent use of cefquinome in lactating small ruminants and could also represent a potential foundation for future trials involving healthy and diseased animals.

## 6. Conclusions

In this research, the disposition of cefquinome after IV and SC administration were well characterised in both sheep and goats, showing rapid elimination and moderate distribution consistent with a beta-lactam antibiotics, although goats exhibited lower volumes of distribution and faster clearance than sheep, resulting in shorter terminal half-lives and reduced systemic exposure. The LA formulation markedly altered absorption kinetics in both species, reducing the absorption rate, increasing the terminal half-lives and sustaining systemic exposure compared with the conventional SC formulation, suggesting its potential utility for optimising therapeutic regimens in small ruminants. However, given the study design, these findings should be interpreted considering the potential contribution of dose-related effects.

Despite the use of lactating animals, cefquinome concentrations in milk were undetectable in goats and very low in sheep, indicating that as with other beta-lactams, its excretion into milk is minimal.

Among the PK/PD indices evaluated, *f*AUC/MIC provided a more robust target for the LA formulation, whereas *f*%T > MIC yielded more conservative MIC values. These differences were reflected in the PK/PD_co_ value estimates, with the LA formulation achieving a higher cut-off and maintaining ≥90% PTA at MICs that exceeded the values achieved with the IV and SC regimens in both species. These findings support the use of both complementary PK/PD indices, particularly in the context of LA formulations, where exposure-based metrics such as fAUC/MIC may enhance the robustness of target attainment analyses. Finally, when compared with published TECOFF, only the LA formulation achieved MICs from pathogens with higher MIC distributions, suggesting that an expanded therapeutic potential for respiratory infections in small ruminants could be obtained with this type of formulation. However, further studies would be needed to evaluate this LA formulation and these PK/PK indices with a larger number of animals in a clinical context taking into account other factors as scores of the clinical symptoms.

## Figures and Tables

**Table 1:** Pharmacokinetic parameters of cefquinome in lactating sheep and lactating goats after IV and SC administration at 2 mg/kg and SC-LA administration at 6 mg/kg.

Parameter	Estimate	IIV (%)	IOV (%)	η (%)	Estimate	IIV (%)	IOV (%)	η (%)
	Lactating sheep				Lactating goats			
F	0.64	16.64		−7.9	0.46	6.38	5.92	38.1
k_a_ (1/h)	0.25	9.07	9.32	36.3	0.53	9.31	7.99	28.1
β_ka_	−1.26				−1.93			
γ	1.53	8.36	7.3	35	1.05	8.56	5.97	39.2
β_γ_	−0.28							
Cl (L/h/kg)	0.18	17.36	-	−7.28	0.12	1.26	-	16
V_1_ (L/kg)	0.16	12.62	-	9.58	0.12	6.36	-	2.75
Q (L/h/kg)	0.17	50.9	-	−2.44	0.067	-	-	
V_2_ (L/kg)	0.12	26.21	-	5.11	0.069	-	-	
Estimate by formulation		Lactating sheep				Lactating goats		
SC		k_a_ (1/h)	0.25	γ	1.5	k_a_ (1/h)	0.53	
SC-LA		k_a_ (1/h)	0.072	γ	1.15	k_a_ (1/h)	0.077	
Error model		A	0.037			B	0.10	
		B	0.047					

F, bioavailability after extravascular administration; k_a_, absorption rate constant; β_ka_, categorical covariate for the absorption rate constant; γ, slope of the absorption phase; β_γ_, categorical covariate for slope of the absorption phase; Cl, clearance; V_1_, volume of distribution at central compartment; Q, intercompartmental clearance; V_2_, volume of distribution at peripheral compartment; IIV and IOV, interindividual and inter-occasion variabilities expressed as percentage of coefficient of variation; η, shrinkage of parameters expressed as percentage; a and b, residual errors.

**Table 2:** Secondary pharmacokinetic parameters of cefquinome in lactating sheep and lactating goats after IV and SC administration at a dose of 2 mg/kg and SC-LA administration at a dose of 6 mg/kg. Data are presented as estimates (CV%).

Parameters	IV	SC	SC-LA	IV	SC	SC-LA
	Lactating Sheep			Lactating Goats		
C_max_ (μg/mL)		1.04 (8.58)	1.11 (6.31)		2.28 (2.83)	1.11 (3.91)
T_max_ (h)		3.6 (15.21)	5.2 (21.07)		1.75 (15.65)	4 (0)
AUC_0_^∞^ (μg/mL∙h)	11.82 (19.53)	6.96 (16.09)	23.02(9.72)	8.11 (5.82)	11.23 (2.74)	17.87 (3.22)
V_ss_ (L/kg)	0.31 (16.76)			0.21 (9.94)		
t_1/2_ (h)	1.66 (31.42)	1.84 (24.24)	10.77 (2.02)	1.44 (8.31)	1.76 (5.97)	7.62 (3.47)
MRT (h)	1.85 (17.48)	5.2 (21.07)	17.47 (2.01)	1.67 (4.8)	3.57 (2.12)	13.27 (1.62)
MAT (h)		3.39 (12.41)	15.61 (1.52)		1.91 (6.89)	11.601 (2.14)

C_max_, the maximum plasma concentration following extravascular administration; T_max_, the time to reach the maximum plasma concentration; AUC_0_^∞^, the area under the concentration–time curve from zero to infinity; V_ss_, volume of distribution at steady state; t_1/2_, the half-life associated with the elimination phase, MRT; mean residence time; MAT, mean absorption time after extravascular administration.

**Table 3:** PK/PD_co_ values of cefquinome in lactating sheep and lactating goats obtained after Monte Carlo simulations.

	Lactating Sheep		
Route/Formulation	IV	SC	SC-LA
Simulated dose regimen	2 mg/kg q24 h 3 days	2 mg/kg q24 h 3 days	6 mg/kg 3 days
*f*T > MIC (40% DI)	ND	0.125	0.125
*f*AUC/MIC (h)	0.125	0.0625	0.25
	**Lactating Goats**		
**Route/Formulation**	**IV**	**SC**	**SC-LA**
Simulated dose regimen	2 mg/kg q24 h 3 days	2 mg/kg q24 h 3 days	6 mg/kg 3 days
*f*T > MIC (40% DI)	0.03125	0.0625	0.125
*f*AUC/MIC (h)	0.125	0.0625	0.25

Data expressed as MIC (μg/mL) to achieve a PK/PD index of cefquinome in the target population greater than a PTA = 90% (probability of target attainment). *f*T > MIC (40% DI), percentage of time that the plasma concentration of unbound cefquinome remains above the MIC during the dosage interval; DI, dosage interval; *f*AUC/MIC, ratio of the area under the plasma concentration–time curve for cefquinome to MIC; ND, not determined.

## Data Availability

The original contributions presented in this study are included in the article/Supplementary Material. Further inquiries can be directed to the corresponding author.

## Supplementary Materials

The following supporting information can be downloaded at: https://www.mdpi.com/article/10.3390/vetsci13060580/s1, Code of NLME model in MLXTRAN^®^ in sheep; code of NLME model in MLXTRAN^®^ in goats; Table S1: Extended pharmacokinetic parameters of cefquinome in lactating sheep after IV and SC administration at 2 mg/kg and SC-LA administration at 6 mg/kg; Table S2: Extended pharmacokinetic parameters of cefquinome in lactating goats after IV and SC administration at 2 mg/kg and SC-LA administration at 6 mg/kg; Table S3: Statistical comparisons of pharmacokinetic parameters of cefquinome in lactating sheep and lactating goats after IV and SC administration at 2 mg/kg and SC-LA administration at 6 mg/kg; Figure S1: Observed versus predicted concentrations. Plots for sheep concentrations; Figure S2: Plots of the population/individual-weighted residuals (PWRES and IWRES) versus predictions/time for sheep concentrations; Figure S3: Box plots for covariate effect for NLME model from sheep concentrations; Figure S4: Observed versus predicted concentrations. Plots for goat concentrations; Figure S5: Plots of the population/individual-weighted residuals (PWRES and IWRES) versus predictions/time for goat concentrations; Figure S6: Box plots for covariate effect for NLME model from goat concentrations; Table S4. Probability of target attainment (PTA) values of simulated dose regimen of cefquinome in lactating sheep. Values calculated with MIC data from 0 to 8 µg/mL using the Monte Carlo simulation (*n* = 10,000); Table S5. Probability of target attainment (PTA) values of simulated dose regimen of cefquinome in lactating goats. Values calculated with MIC data from 0 to 8 µg/mL using the Monte Carlo simulation (*n* = 10,000).
