# Supplementary material for: Pharmacokinetic/Pharmacodynamic Modelling of Cefquinome in Lactating Sheep and Lactating Goats After Intravenous, Subcutaneous and Long-Acting Administrations
Source: Vet Sci. 2026 Jun 13;13(6):580. doi: 10.3390/vetsci13060580 (PMC13307860; doi:10.3390/vetsci13060580)
Supplement: Supplementary file 1 [file vetsci-13-00580-s001.zip › vetsci-4322106-supplementary.pdf]

Supplementary Material

CONTENTS

1. Code of NLME model in MLXTRAN® code in sheep.
2. Code of NLME model in MLXTRAN® code in goats.
3. Table S1. Extended pharmacokinetic parameters of cefquinome in lactating sheep after IV and SC administration at 2 mg/kg, and SC LA administration at 6 mg/kg.
4. Table S2. Extended pharmacokinetic parameters of cefquinome in lactating goats after IV and SC administration at 2 mg/kg, and SC LA administration at 6 mg/kg.
5. Table S3. Statistical comparisons of pharmacokinetic parameters of cefquinome in lactating sheep and lactating goats after IV and SC administration at 2 mg/kg, and SC LA administration at 6 mg/kg.
6. Figure S1: Observed versus predicted concentrations. plots for sheep concentrations
7. Figure S2: Plots of the population/individual-weighted residuals (PWRES and IWRES) versus predictions/time for sheep concentrations
8. Figure S3: Box plots for covariate effect for NLME model from sheep concentrations.
9. Figure S4: Observed versus predicted concentrations. plots for goats concentrations
10. Figure S5: Plots of the population/individual-weighted residuals (PWRES and IWRES) versus predictions/time for goats concentrations
11. Figure S6: Box plots for covariate effect for NLME model from goats concentrations.
12. Table S4. Probability of target attainment (PTA) values of simulated dose regimen of cefquinome in lactating sheep. Values calculated with MIC data from 0 to 8 μg/ml using the Monte Carlo simulation (n = 10000). Both f%T>MIC and fAUC/MIC were used.
13. Table S5. Probability of target attainment (PTA) values of simulated dose regimen of cefquinome in lactating goats. Values calculated with MIC data from 0 to 8 μg/ml using the Monte Carlo simulation (n = 10000). Both f%T>MIC and fAUC/MIC were used.

Code of NLME model in MLXTRAN® Sheep

DESCRIPTION: BICOMPARTMENTAL MODEL FOR PLASMA PARAMETRIZED BY CL AND Q, ABSORPTION MODEL WITH WEIBOULL MODEL. Sheep

```
[LONGITUDINAL]

input = {F, ka, gamma, Cl, V1, Q2, V2}

PK:

depot(adm=1, target=A1) ; IV administration

depot(adm=2, target=A3, p=F) ; SC administration (reference/polimeric)


EQUATION:

odeType = stiff

t_0=0

A1_0=0 ; IV central compartment

A2_0=0 ; Peripheral compartment 1

A3_0=0 ; SC administration (reference/polimeric)


; Parameter transformations

k1 = Cl/V1

k12 = Q2/V1

k21 = Q2/V2


fu=0.8435


Weibull = ka*gamma*((max(t,0)*ka)^(gamma-1))


; ODE system

ddt_A1 = Weibull*A3 - Cl/V1*fu*A1 - Q2/V1*fu*A1 + Q2/V2*A2
```

```
ddt_A2 = Q2/V1*fu*A1 - Q2/V2*A2

ddt_A3 = - Weibull*A3


; concentrations

C1 = fu*A1/V1 ; Free plasma concentrations at central compartment

C2 = A2/V2 ; Peripheral concentrations 2


; Secondary parameters

AUC_0 = 0

ddt_AUC = fu*A1/V1

AUC24_0 = 0

if(t < 24)

  dAUC24 = 1/V1 * fu*A1

else

  dAUC24 = 0

end

ddt_AUC24 = dAUC24

Vss = V1 + V2

kel = Cl/V1

k1 = Cl/V1

k12 = Q2/V1

k21 = Q2/V2

Beta = 0.5 *(kel + k12 + k21 -((k12 + k21 + kel)^2 - (4 * k21 * kel))^0.5)

HLbeta = 0.693/Beta



OUTPUT:

output = {C1}

table = {Weibull, AUC, AUC24, Vss, HLbeta}
```

Code of NLME model in MLXTRAN®. Goats

DESCRIPTION: BICOMPARTMENTAL MODEL FOR PLASMA PARAMETRIZED BY CL AND Q, ABSORPTION MODEL WITH WEIBOULL MODEL. GOATS

```
[LONGITUDINAL]

input = {F, ka, gamma, Cl, V1, Q2, V2}

PK:

depot(adm=1, target=A1) ; IV administration

depot(adm=2, target=A3, p=F) ; SC administration (reference/polimeric)


EQUATION:

odeType = stiff

t_0=0

A1_0=0 ; IV central compartment

A2_0=0 ; Peripheral compartment 1

A3_0=0 ; SC administration (reference/polimeric)


; Parameter transformations

k1 = Cl/V1

k12 = Q2/V1

k21 = Q2/V2


fu=0.8560


Weibull = ka*gamma*((max(t,0)*ka)^(gamma-1))


; ODE system

ddt_A1 = Weibull*A3 - Cl/V1*fu*A1 - Q2/V1*fu*A1 + Q2/V2*A2
```

```
ddt_A2 = Q2/V1*fu*A1 - Q2/V2*A2

ddt_A3 = - Weibull*A3


; concentrations

C1 = fu*A1/V1 ; Free plasma concentrations at central compartment

C2 = A2/V2 ; Peripheral concentrations 2


; Secondary parameters

AUC_0 = 0

ddt_AUC = fu*A1/V1

AUC24_0 = 0

if(t < 24)

  dAUC24 = 1/V1 * fu*A1

else

  dAUC24 = 0

end

ddt_AUC24 = dAUC24

Vss = V1 + V2

kel = Cl/V1

k1 = Cl/V1

k12 = Q2/V1

k21 = Q2/V2

Beta = 0.5 *(kel + k12 + k21 -((k12 + k21 + kel)^2 - (4 * k21 * kel))^0.5)

HLbeta = 0.693/Beta



OUTPUT:

output = {C1}

table = {Weibull, AUC, AUC24, Vss, HLbeta}
```

Table S1.

Extended pharmacokinetic parameters of cefquinome in lactating sheep after IV and SC administration at 2 mg/kg, and SC-P407-CMC administration at 6 mg/kg.

	VALUE		LINEARIZATION				BOOTSTRAP (N=200)		
			S.E.	R.S.E.(%)	P2.5	P97.5	P2.5	MEDIAN	P97.5
**Fixed Effects**									
F_pop	0.64		0.051	8.07	0.53	0.73	0.53	0.66	0.73
ka_pop	0.25		0.016	6.31	0.22	0.29	0.23	0.25	0.26
beta_ka_tFORMULATION_G_C	-1.26		0.07	5.54	-1.4	-1.12	-1.32	-1.26	-1.19
gamma_pop	1.53		0.081	5.28	1.38	1.7	1.44	1.51	1.63
beta_gamma_tFORMULATION_G_C	-0.29		0.051	17.9	-0.39	-0.19	-0.36	-0.27	-0.2
Cl_pop	0.18		0.014	7.87	0.15	0.2	0.16	0.18	0.2
V1_pop	0.16		0.0093	5.99	0.14	0.18	0.13	0.16	0.17
Q2_pop	0.17		0.04	24.2	0.11	0.26	0.097	0.18	0.43
V2_pop	0.12		0.016	12.7	0.097	0.16	0.1	0.13	0.16
**Fixed Effects by Category**									
ka_tFORMULATION_G_A_B	0.25		0.016	6.31	0.22	0.29			
ka_tFORMULATION_G_C	0.072		0.0047	6.61	0.063	0.082			
gamma_tFORMULATION_G_A_B	1.53		0.081	5.28	1.38	1.7			
gamma_tFORMULATION_G_C	1.15		0.054	4.68	1.05	1.26			
**Standard Deviation of the Random Effects**									
	Value	C.V.(%)							
omega_F	0.47	16.66	0.17	36.4	0.25	0.91	0.02	0.44	0.59
omega_ka	0.09	9.03	0.055	61.5	0.033	0.24	0.087	0.09	0.092
omega_gamma	0.084	8.4	0.041	49.3	0.036	0.19	0.081	0.083	0.086
omega_Cl	0.17	17.47	0.056	32.2	0.096	0.31	0.016	0.17	0.23
omega_V1	0.12	11.99	0.046	38.7	0.06	0.24	0.0033	0.12	0.16
omega_Q2	0.47	49.92	0.18	37.4	0.24	0.92	0.012	0.48	0.66
omega_V2	0.25	25.43	0.098	39	0.12	0.5	0.013	0.23	0.4
gamma_F	0.18	6.39	0.087	49	0.077	0.41	0.16	0.2	0.26
gamma_ka	0.095	9.55	0.04	41.9	0.046	0.2	0.085	0.094	0.097
gamma_gamma	0.073	7.26	0.029	40	0.036	0.15	0.063	0.071	0.074
**Error Model Parameters**									
a	0.037		0.0046	12.5	0.029	0.047	0.026	0.036	0.041
b	0.048		0.007	14.5	0.036	0.064	0.039	0.048	0.061

Table S2.

Extended pharmacokinetic parameters of cefquinome in lactating goats after IV and SC administration at 2 mg/kg, and SC LA administration at 6 mg/kg.

	VALUE		LINEARIZATION				BOOTSTRAP (N=200)		
			S.E.	R.S.E.(%)	P2.5	P97.5	P2.5	MEDIAN	P97.5
**Fixed Effects**									
F_pop	0.46		0.016	3.49	0.43	0.49	0.45	0.46	0.48
ka_pop	0.53		0.027	5.12	0.48	0.59	0.51	0.53	0.55
beta_ka_tFORMULATION_G_C	-1.93		0.05	2.6	-2.03	-1.83	-1.96	-1.92	-1.9
gamma_pop	1.05		0.039	3.71	0.97	1.13	1.03	1.05	1.06
Cl_pop	0.12		0.0016	1.31	0.12	0.13	0.12	0.13	0.13
V1_pop	0.12		0.005	4.01	0.11	0.13	0.11	0.13	0.13
Q2_pop	0.067		0.0082	12.2	0.053	0.085	0.053	0.066	0.089
V2_pop	0.069		0.0042	6.1	0.061	0.078	0.058	0.068	0.084
**Fixed Effects by Category**									
ka_tFORMULATION_G_A_B	0.53		0.027	5.12	0.48	0.59			
ka_tFORMULATION_G_C	0.077		0.0035	4.52	0.071	0.084			
**Standard Deviation of the Random Effects**									
	Value	C.V.(%)							
omega_F	0.12	6.38	0.058	49.4	0.051	0.27	0.12	0.12	0.12
omega_ka	0.093	9.31	0.041	44	0.043	0.2	0.091	0.092	0.095
omega_gamma	0.085	8.56	0.032	37.7	0.043	0.17	0.085	0.085	0.086
omega_Cl	0.013	1.26	0.016	**124**	0.0027	0.058	0.00081	0.0083	0.023
omega_V1	0.063	6.36	0.027	43.1	0.03	0.14	0.0037	0.026	0.099
gamma_F	0.11	5.92	0.042	38.6	0.055	0.22	0.11	0.11	0.11
gamma_ka	0.08	7.99	0.029	36.2	0.042	0.15	0.075	0.077	0.086
gamma_gamma	0.06	5.97	0.02	33.1	0.033	0.11	0.055	0.06	0.062
**Error Model Parameters**									
b	0.1		0.0052	5.23	0.09	0.11	0.087	0.099	0.1

Table S3. Statistical comparisons of pharmacokinetic parameters of cefquinome in lactating sheep and lactating goats after IV and SC administration at 2 mg/kg, and SC LA administration at 6 mg/kg.

Comparisons of parameters between lactating sheep and lactating goats								
Parameter	F_{SC}	$F_{SC\ LA}$	k_{aSC}	$k_{aSC\ LA}$	γ_{SC}	$\gamma_{SC\ LA}$		
p value	$2.90{\cdot}10^{-06}$	$3.38{\cdot}10^{-03}$	$3.56{\cdot}10^{-06}$	$1.78{\cdot}10^{-08}$	$2.50{\cdot}10^{-07}$	$7.70{\cdot}10^{-04}$		
Observations	a	a	b	b	b	a		
Parameter	C_{maxSC}	$C_{maxSC\ LA}$	T_{maxSC}	$T_{maxSC\ LA}$	V_{ss}	Cl	$t_{1/2}$	
p value	$5.31{\cdot}10^{-10}$	0.97	$4.51{\cdot}10^{-06}$	$2.30{\cdot}10^{-02}$	$5.98{\cdot}10^{-04}$	$9.95{\cdot}10^{-03}$	0.35	
Observations	c	nd	d	d	a	a	nd	
Comparisons of parameters after subcutaneous administrations in lactating sheep								
Parameter	F	k_a	γ	MAT	$t_{1/2}$	C_{max}	T_{max}	
p value	0.73	$3.5{\cdot}10^{-09}$	$7.10{\cdot}10^{-04}$	$1.30{\cdot}10^{-07}$	$8.00{\cdot}10^{-07}$	0.62	$3.5{\cdot}10^{-02}$	
Observations	Nd	e	e	f	f	nd	f	
Comparisons of parameters after subcutaneous LA administrations in lactating goats								
Parameter	F	k_a	γ	MAT	$t_{1/2}$	$t_{1/2\ post\ hoc}$	C_{max}	T_{max}
p value	0.29	$1.78{\cdot}10^{-08}$	0.072	$1.92{\cdot}10^{-09}$	$9.08{\cdot}10^{-09}$	$5.1{\cdot}10^{-03}$	$7.32{\cdot}10^{-07}$	$5.61{\cdot}10^{-07}$
Observations	nd	e	nd	f	f	g	h	f

F_{SC}, bioavailability after subcutaneous administration; $F_{SC\text{-}P407\text{-}CMC}$, bioavailability after subcutaneous long acting administration; k_{aSC}, absorption rate constant after subcutaneous administration; $k_{aSC\text{-}P407\text{-}CMC}$, absorption rate constant after subcutaneous long acting administration; γ_{SC}, slope of the absorption phase after subcutaneous administration; $\gamma_{SC\text{-}P407\text{-}CMC}$, slope of the absorption phase after subcutaneous long acting administration; V_{ss}, volume of distribution at steady state; Cl, clearance; $t_{1/2}$,the half-life associated with the elimination phase; MAT, mean absorption time after extravascular administration; $t_{1/2}$ *post hoc* is; comparison of half-lives as second post-test.

a, higher in lactating sheep; b, lower in lactating sheep; c, higher in lactating goats; d, longer in lactating sheep; e, lower in LA formulation; f, longer in LA formulation; g, longer in SC formulation versus IV formulation; h, higher for SC formulation. nd; no differences were detected. Significant difference at $p < 0.05$.

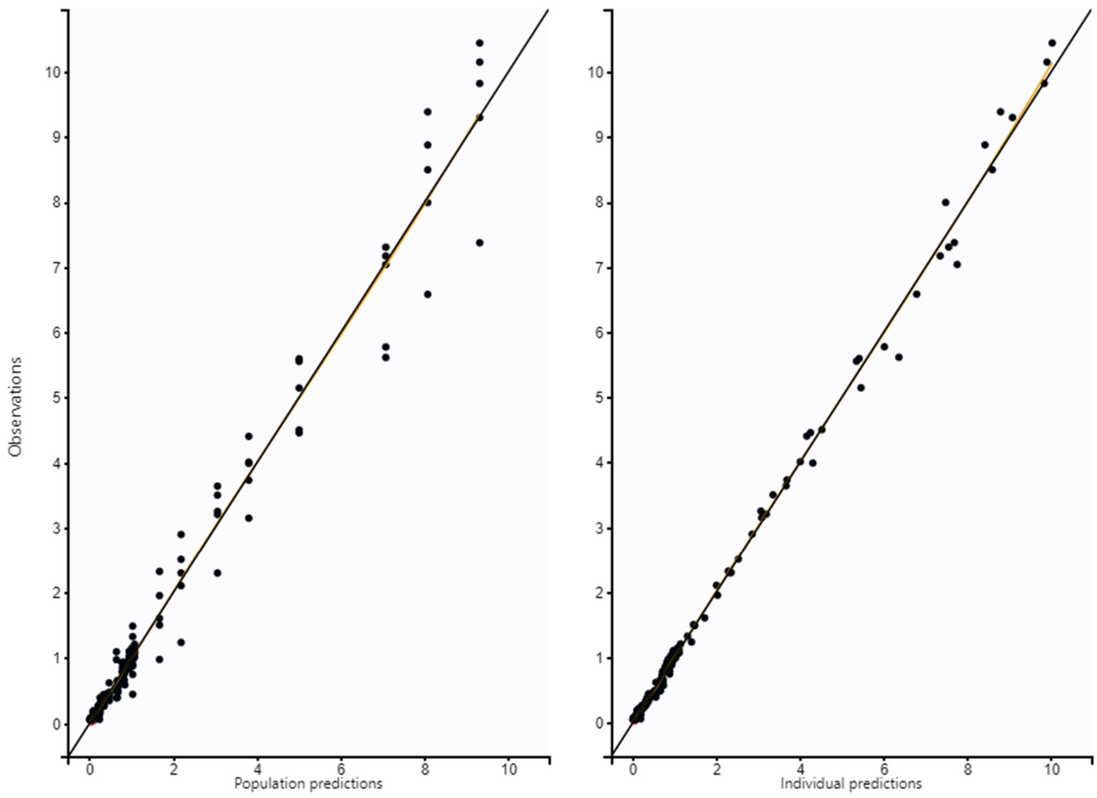

Figure S1.

Observed versus predicted concentrations plots for sheep concentrations in plasma. Left panel shows the observations versus populations predictions. The right panel shows the observations versus individual predictions.

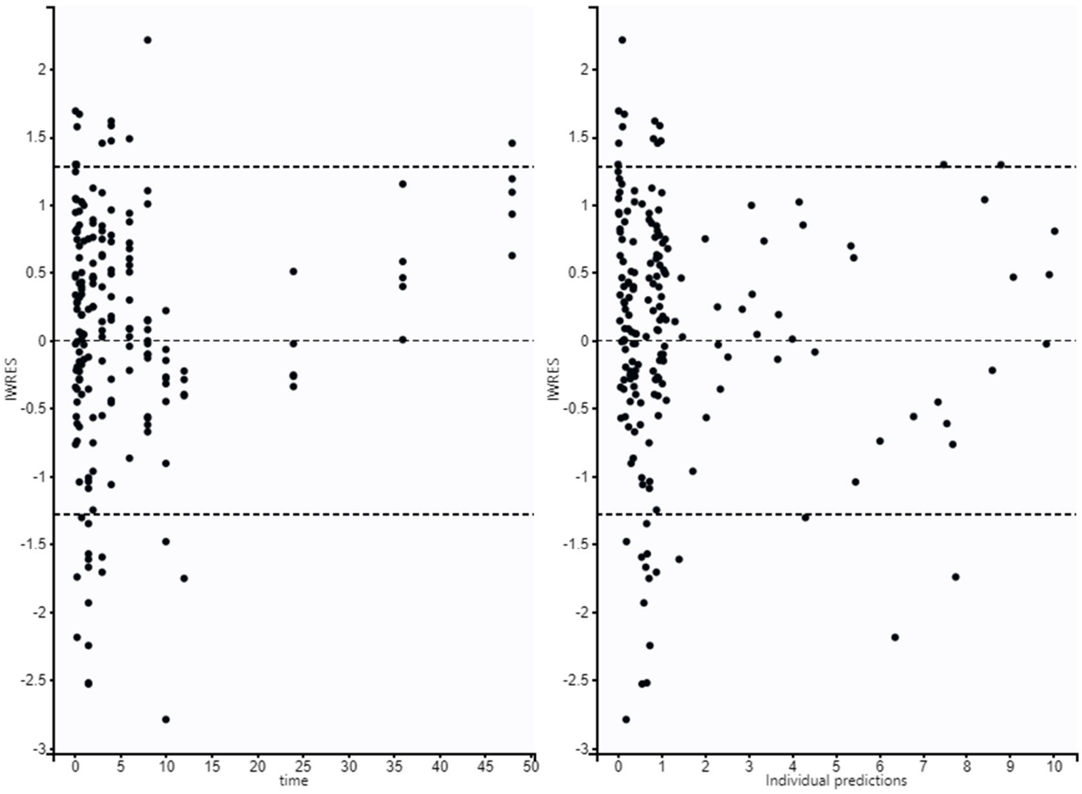

Figure S2

Plots of the population/individual-weighted residuals (PWRES and IWRES) versus predictions/time for sheep plasma concentration

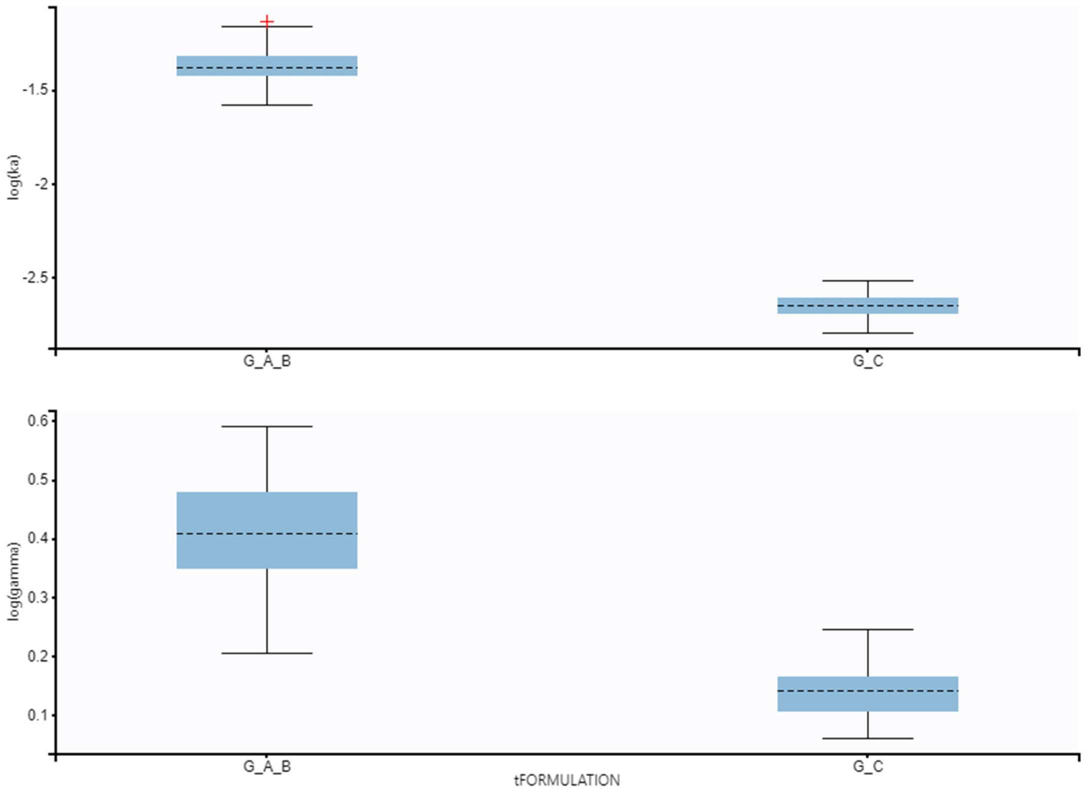

Figure S3: Box plots for covariate effect of the LA formulation in the typical values of the absorption rate and gamma value in sheep.

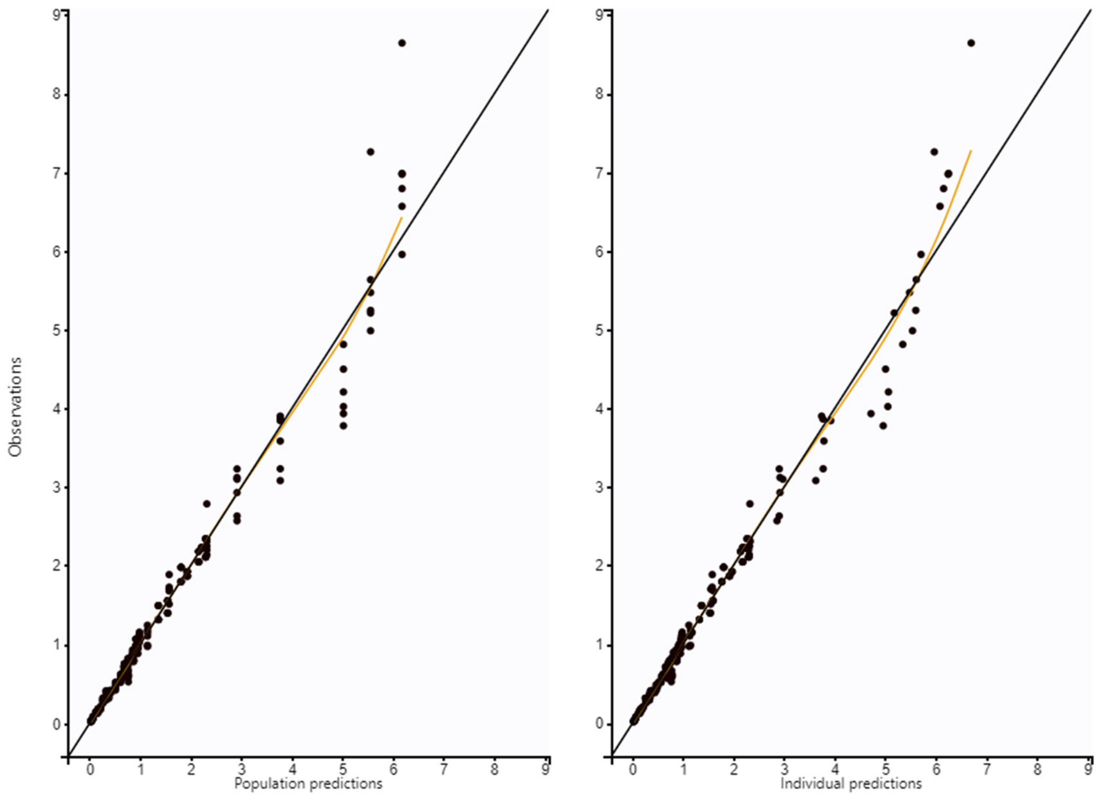

Figure S4.

Observed versus predicted concentrations plots for goat concentrations in plasma. Left panel shows the observations versus populations predictions. The right panel shows the observations versus individual predictions.

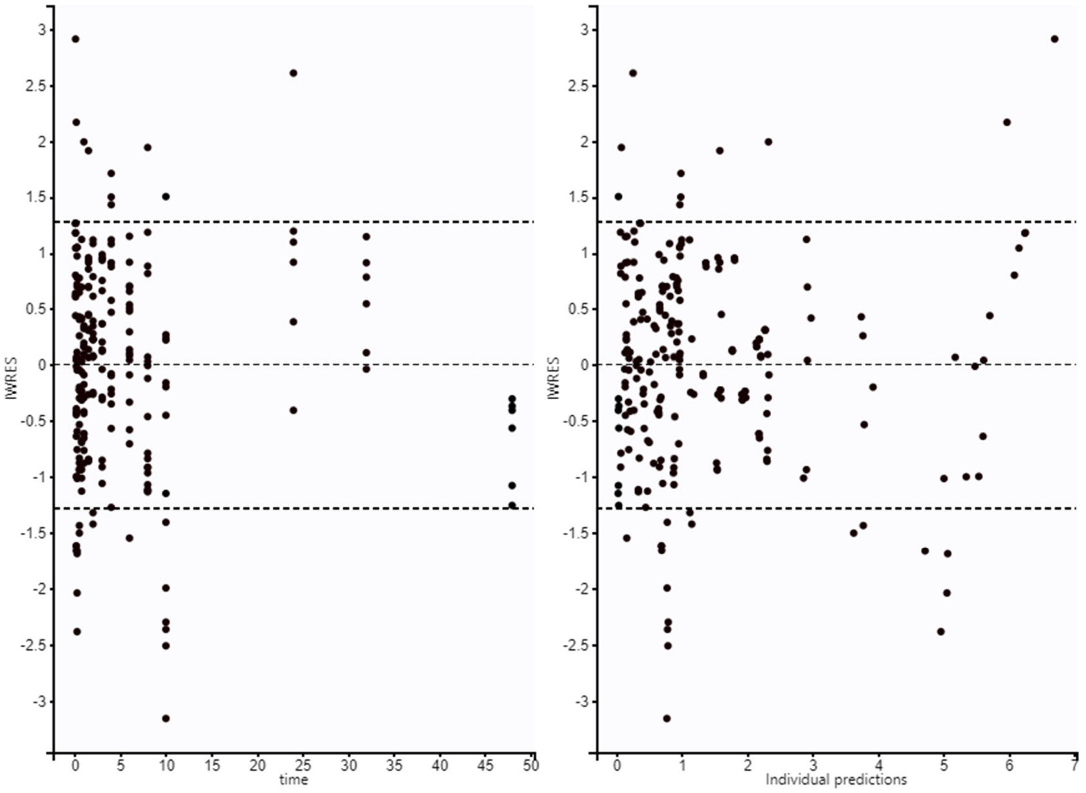

Figure S5

Plots of the population/individual-weighted residuals (PWRES and IWRES) versus predictions/time for goat plasma concentration

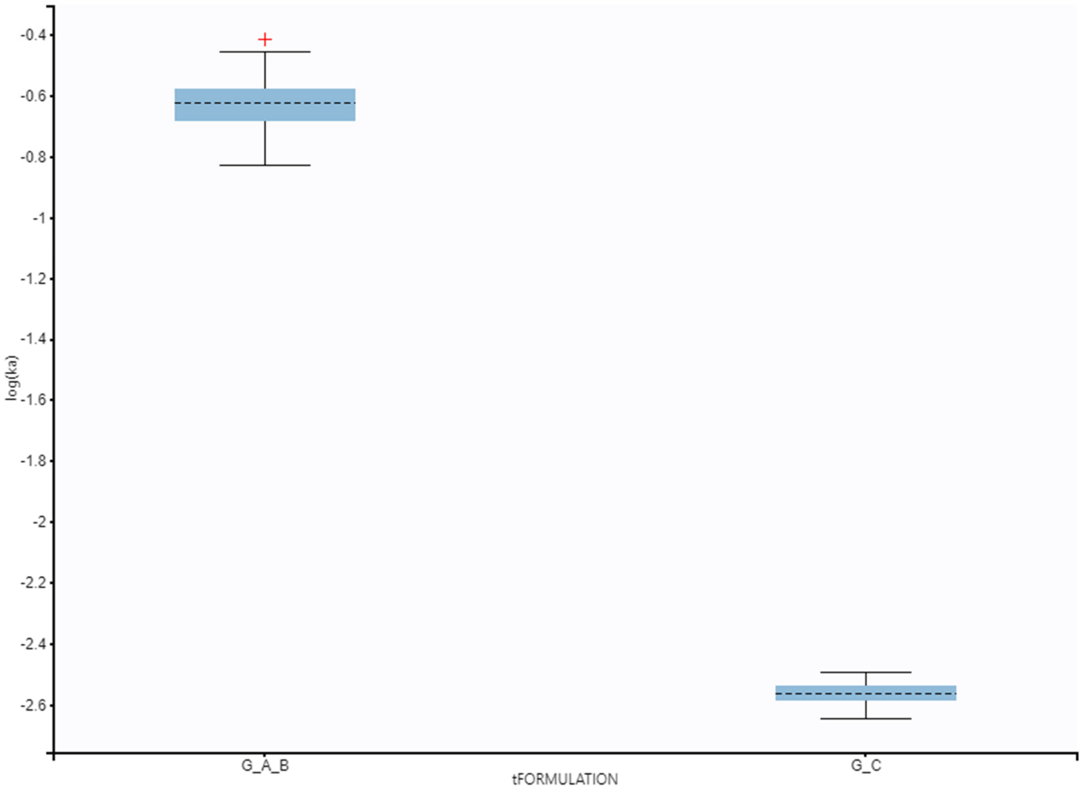

Figure S6: Box plots for covariate effect of the LA formulation in the typical values of the absorption rate and in goats.

Table S4.

Probability of target attainment (PTA) values of simulated dose regimen of cefquinome in lactating sheep. Values calculated with MIC data from 0 to 8 μg/ml using the Monte Carlo simulation (n = 10000). Both *f*%T>MIC and *f*AUC/MIC were used.

PK/PD index	*f*T>MIC	*f*T>MIC	*f*T>MIC	*f*AUC/MIC	*f*AUC/MIC	*f*AUC/MIC
Dose regimen	IV	SC	SC LA	IV	SC	SC LA
MIC	% of PTA	% of PTA	% of PTA	% of PTA	% of PTA	% of PTA
0	88.2	100	100	100	100	100
0.008	83.2	100	100	100	100	100
0.016	77.4	100	100	100	100	100
0.031	67.8	100	100	100	100	100
0.0625	45.6	99	100	100	100	100
0.125	15.2	91.4	93	92.37	58.2	100
0.25	0	25	27	46.18	29.1	100
0.5	0	0	0	23.09	14.55	43.59
1	0	0	0	11.55	7.27	21.79
2	0	0	0	5.77	3.64	10
4	0	0	0	2.1	0	5.45
8	0	0	0	0	0	2.72

Table S5.

Probability of target attainment (PTA) values of simulated dose regimen of cefquinome in lactating goats. Values calculated with MIC data from 0 to 8 µg/ml using the Monte Carlo simulation (n = 10000). Both *f*%T>MIC and *f*AUC/MIC were used.

PK/PD index	*f*T>MIC	*f*T>MIC	*f*T>MIC	*f*AUC/MIC	*f*AUC/MIC	*f*AUC/MIC
Dose regimen	IV	SC	SC-LA	IV	SC	SC-LA
MIC	% of PTA	% of PTA	% of PTA	% of PTA	% of PTA	% of PTA
0	100	100	100	100	100	100
0.008	100	100	100	100	100	100
0.016	100	100	100	100	100	100
0.031	100	100	100	100	100	100
0.0625	53.2	98.8	100	100	100	100
0.125	0	32.4	95.6	100	59.1	100
0.25	0	0	3.6	64.13	29.55	100
0.5	0	0	3.6	32.06	14.77	44.08
1	0	0	0	16.03	7.39	22.04
2	0	0	0	8.02	4.01	11.02
4	0	0	0	4.01	1.85	5.51
8	0	0	0	2	0.25	2.75
